# Burden of smoking in Asia-Pacific countries

**DOI:** 10.18332/tid/133633

**Published:** 2021-04-15

**Authors:** Vivian W. Y. Lee, Anna Li, Joyce T. S. Li

**Affiliations:** 1 Centre for Learning Enhancement and Research, The Chinese University of Hong Kong, Hong Kong; 2 School of Pharmacy, Faculty of Medicine, The Chinese University of Hong Kong, Hong Kong

**Keywords:** smoking cessation, cigarette, Asia, healthcare expenditures, burden of smoking

## Abstract

**INTRODUCTION:**

Smoking is a modifiable risk factor for many diseases. The public should recognize the impact of smoking on their health and their wealth. The current study aimed to evaluate the cost burden of smoking to target Asia-Pacific countries.

**METHODS:**

The current study estimated the annual spending and lifetime spending of smokers in the target Asia-Pacific countries (Hong Kong, Malaysia, Thailand, South Korea, Singapore, and Australia) on purchasing cigarettes, as well as predicted the revenue that could be generated if smokers spent the money on investment instead of buying cigarettes. Smokers’ spending on cigarettes and the potential revenue generated from investment were estimated based on the selling prices of cigarettes, Standards & Poor’s 500 Index, and life expectancies of smokers. Data were extracted from reports released by the World Health Organization or government authorities.

**RESULTS:**

The annual expenses (in US$) on purchasing one pack of cigarettes, in decreasing order, were: Australia ($5628.30), Singapore ($3777.75), Hong Kong ($2799.55), Malaysia ($1529.35), South Korea ($1467.30), and Thailand ($657.00). The lifetime spending on purchasing one pack of cigarettes each day were: Australia ($308993.67), Singapore ($207398.48), Hong Kong ($151735.61 for male and $166853.18 for female), South Korea ($80261.31), Malaysia ($72338.26), and Thailand ($31207.50).

**CONCLUSIONS:**

The cost burden of smoking is high from a smoker’s perspective. Smokers should recognize the high economic burden and quit smoking to enjoy better health and wealth.

## INTRODUCTION

Smoking is a well-established modifiable risk factor for many chronic diseases, including cardiovascular diseases, cerebrovascular diseases, and respiratory diseases. Globally, over 8 million people are killed by tobacco each year. More than 7 million deaths are due to direct tobacco use while about 1.2 million deaths are due to secondhand smoke exposure^1^.

Asian countries are in the early stages of the tobacco smoking epidemic. The projected prevalence of consuming any tobacco product among males aged ≥15 years in 2020 was: 40.2% in Malaysia, 39.7% in Thailand, 46.9% in South Korea, 28.3% in Singapore, 14.3% in Australia, and 10.0% in Hong Kong^2^. The World Health Organization Framework Convention on Tobacco Control recommends that all Asian countries implement comprehensive tobacco control policies such as raising tobacco taxes and prices, and implementing smoke-free laws, to reduce smoking prevalence in Asia^3^. Tobacco is also the leading cause of cancer in Australia, which contributes to 22% of the cancer burden^4^. The Australian government has implemented a tobacco advertising ban, plain packaging law, law on smoking in public, and age limit in purchasing cigarettes, to reduce tobacco use.

Smoking would undoubtedly impose far-reaching consequences to human health. Traditionally, authorities tend to promote smoking cessation by emphasizing the undesirable impact of smoking on health. Nevertheless, the direct economic impact of smoking on smokers is not often discussed. While some studies have attempted to predict the healthcare burden imposed on society due to smoking^5^, relatively few studies have estimated the economic impact of smoking on individual smokers. This study investigated the financial impact of smoking on an individual level to determine the burden of smoking in six Asia-Pacific countries, namely, Hong Kong, Malaysia, Thailand, South Korea, Singapore, and Australia. We aimed to estimate smokers’ spending on cigarettes and show smokers that giving up smoking can bring them better wealth.

## METHODS

The current study estimated the annual spending and lifetime spending of smokers on purchasing cigarettes, as well as predicted the revenue that could be generated if smokers spent the money on investment instead of buying cigarettes.

We estimated the annual spending and lifetime spending of smokers on purchasing cigarettes if they smoked 1 pack, 1.5 packs, 2 packs or 3 packs of cigarettes each day starting from the age of 18 years. Inflation or changes in cigarette prices over time were not taken into account in the above estimations. The selling price of cigarettes in Hong Kong were cited from government reports^6^, while those of target Asia-Pacific countries were determined from data released by the World Health Organization (WHO)^7^. Citizens’ average life expectancies at birth were used to estimate their lifetime spending on cigarettes. Data for Hong Kong were from government reports, while those of the target Asia-Pacific countries were from data released by WHO^8,9^.

In addition, references were made to Standards & Poor’s 500 Index (S&P 500) to estimate the revenue smokers could return on retirement if they used the money to make investments instead of buying cigarettes. S&P 500, which was a widely reported stock market index that measured the stock performance of 500 companies in the United States, was chosen to avoid bias towards any country in this study.

Amount returned from investment in 2019 was calculated from:

$$Amount=a(\frac{1-r^{\underline{n}}}{1-r})$$

where *a* is the annual expense, *r* the annualized growth rate of the S&P 500, and n the period. The formula was used to predict the amount that could be generated when the smokers retired.

## RESULTS

Table 1 shows the out-of-pocket amount smokers spent on purchasing cigarettes in Hong Kong, Malaysia, Thailand, South Korea, Singapore, and Australia. Prices listed in Table 1 refer to the retail price of one pack of the most-sold brand of cigarettes in each country in 2018, as reported by WHO. In general, cigarette smoking would reduce average life expectancy by about 10 years. Therefore, we deducted 10 years from average life expectancies at birth when calculating the lifetime expenses on cigarettes. Annual expenses and lifetime expenses (starting from the age of 18 years) were calculated as 365 days per year. Retail prices of one pack of cigarettes were $7.67 in Hong Kong, $4.19 in Malaysia, $1.80 in Thailand, $4.02 in South Korea, $10.35 in Singapore, and $15.42 in Australia. Annual expenses of purchasing one pack of cigarettes per day would be $2799.55 in Hong Kong, $1529.35 in Malaysia, $657.00 in Thailand, $1467.30 in South Korea, $3777.75 in Singapore, and $5628.30 in Australia.

**Table 1:** Financial impact of cigarettes on individuals and society in target Asia-Pacific countries

*Impact*	*Hong Kong*				*Malaysia*				*Thailand*			
Cost per pack (US$) ^6,7^	7.67				4.19				1.80			
Average life expectancy (years) ^8,9^	Male: 82.2 (Female: 87.6)				75.3				75.5			
Average life expectancy for smokers (years)	Male: 72.2 (Female: 77.6)				65.3				65.5			
Number of packs per day	1	1.5	2	3	1	1.5	2	3	1	1.5	2	3
Annual expenses for one pack per day (US$)	2799.55 (2799.55)	4199.33 (4199.33)	5599.10 (5599.10)	8398.65 (8398.65)	1529.35	2294.03	3058.70	4588.05	657.00	985.50	1314.00	1971.00
Lifetime expenses for one pack per day (US$)	151735.61 (166853.18)	227603.69 (250280.07)	303471.22 (333706.36)	455206.83 (500559.54)	72338.26	108507.38	144676.51	217014.77	31207.50	46811.25	62415.00	93622.50
S&P 500 CAGR	7.39%				7.39%				7.39%			
Total amount at retirement if invested in S&P 500 index (US$)	718819.55	1078230.61	1437639.10	2156458.66	392679.78	589019.67	785359.56	1178039.34	168692.98	253039.48	337385.97	506078.95
***Countries***	***South Korea***				***Singapore***				***Australia***			
Cost per pack (US$) ^7^	4.02				10.35				15.42			
Average life expectancy (years) ^9^	82.7				82.9				82.9			
Average life expectancy for smokers (years)	72.7				72.9				72.9			
Number of packs per day	1	1.5	2	3	1	1.5	2	3	1	1.5	2	3
Annual expenses for one pack per day (US$)	1467.30	2200.95	2934.60	4401.90	3777.75	5666.63	7555.50	11333.25	5628.30	8442.45	11256.60	16884.90
Lifetime expenses for one pack per day (US$)	80261.31	120391.97	160522.62	240783.93	207398.48	311097.71	414796.95	622195.43	308993.67	463490.51	617987.34	926981.01
S&P 500 CAGR	7.39%				7.39%				7.39%			
Total amount at retirement if invested in S&P 500 index (US$)	376747.67	565121.50	753495.33	1130243.00	969984.66	1454976.99	1939969.33	2909953.99	1445136.57	2167704.85	2890273.14	4335409.71

The average life expectancy in Hong Kong was cited from a local government report. Data on male and female were reported separately. The average life expectancies in other countries were cited from WHO, which reported the data on both sexes as a whole.

Furthermore, we estimated the revenue smokers could gain if they invested the money spent on cigarettes. Assuming a person started investing at the age of 18 years and retired at the age of 60 years, it would be equivalent to an investment period of 42 years. The annualized growth rate of the S&P 500 between January 1976 and December 2018 was 7.39%, adjusted for inflation^10^. Table 1 shows the estimated amount that would be returned when a person retired if he invested the money of buying 1 pack, 1.5 packs, 2 packs, or 3 packs of cigarettes.

## DISCUSSION

Among the six countries, the selling price of cigarettes was highest in Australia, Singapore, and Hong Kong. Annual expenses to purchase one pack of cigarettes each day were $5628.30 in Australia, $3777.75 in Singapore, and $2799.55 in Hong Kong. This spending corresponds to a substantial portion of a citizen’s income. It was reported that the all-employees average weekly total earnings in Australia was AUD 1238.30 ($854.43) in May 2019^11^, the median monthly household income from work per household member in Singapore was SGD 2792 ($2066.08) in 2018^12^, and the median monthly employment earnings of employed persons (excluding foreign domestic helpers) in Hong Kong was HKD 18900 ($2457.00) in the third quarter of 2019^13^. In other words, smokers were spending annually more than the money they earned in one month to smoke one pack of cigarettes each day.

The economic impact of smoking would be more obvious if smokers considered the potential profit generated from the investment. On average, each smoker in Hong Kong smokes 12.4 cigarettes per day, which is equivalent to an annual expense of $1735.72^14^. Investing this amount could generate $445667.87, which is equivalent to 15.11 years of working, and it is close to the market price of a 533 square feet private housing apartment in Hong Kong. If smokers invest the cost of one pack of cigarettes per day in the stock market, an amount equivalent to 24.38 years of working could be generated and smokers could purchase an 820 square feet private apartment in Hong Kong. Smokers should recognize the financial impact of smoking and the benefit of smoking cessation.

The current study illustrates the direct expenses of smokers on cigarette smoking. Nonetheless, the economic burden of smoking to individual smokers is in fact much heavier if indirect expenditures such as healthcare costs for managing smoking-related diseases and productivity loss from taking smoking breaks and absenteeism are taken into consideration. Although smoking is a well-proven modifiable risk factor for multiple diseases, citizens may not fully recognize the health impacts of smoking. For example, it was reported that citizens were aware that smoking would increase the risk of lung cancer, but they were less aware of the increase in the risk of cardiovascular diseases or stroke^15^. It indicates that there are ramifications of smoking of which smokers are not fully aware. Smokers should be educated that smoking cessation can alleviate the economic burden on both individuals and society.

## CONCLUSIONS

Smoking imposes a huge financial burden on smokers. Smokers should recognize the economic impact of smoking and reduce tobacco use to optimize the value of their money.
